# SYNCRIP facilitates porcine parvovirus viral DNA replication through the alternative splicing of NS1 mRNA to promote NS2 mRNA formation

**DOI:** 10.1186/s13567-021-00938-6

**Published:** 2021-05-25

**Authors:** Songbiao Chen, Bichen Miao, Nannan Chen, Caiyi Chen, Ting Shao, Xuezhi Zhang, Lingling Chang, Xiujuan Zhang, Qian Du, Yong Huang, Dewen Tong

**Affiliations:** College of Veterinary Medicine, Northwest A&F University, Yangling, China

**Keywords:** PPV, SYNCRIP, NS1 mRNA, Alternative splicing, NS2 mRNA

## Abstract

Porcine Parvovirus (PPV), a pathogen causing porcine reproductive disorders, encodes two capsid proteins (VP1 and VP2) and three nonstructural proteins (NS1, NS2 and SAT) in infected cells. The PPV NS2 mRNA is from NS1 mRNA after alternative splicing, yet the corresponding mechanism is unclear. In this study, we identified a PPV NS1 mRNA binding protein SYNCRIP, which belongs to the hnRNP family and has been identified to be involved in host pre-mRNA splicing by RNA-pulldown and mass spectrometry approaches. SYNCRIP was found to be significantly up-regulated by PPV infection in vivo and in vitro. We confirmed that it directly interacts with PPV NS1 mRNA and is co-localized at the cytoplasm in PPV-infected cells. Overexpression of SYNCRIP significantly reduced the NS1 mRNA and protein levels, whereas deletion of SYNCRIP significantly reduced NS2 mRNA and protein levels and the ratio of NS2 to NS1, and further impaired replication of the PPV. Furthermore, we found that SYNCRIP was able to bind the 3′-terminal site of NS1 mRNA to promote the cleavage of NS1 mRNA into NS2 mRNA. Taken together, the results presented here demonstrate that SYNCRIP is a critical molecule in the alternative splicing process of PPV mRNA, while revealing a novel function for this protein and providing a potential target of antiviral intervention for the control of porcine parvovirus disease.

**Supplementary Information:**

The online version contains supplementary material available at 10.1186/s13567-021-00938-6.

## Introduction

**Figure 1:**
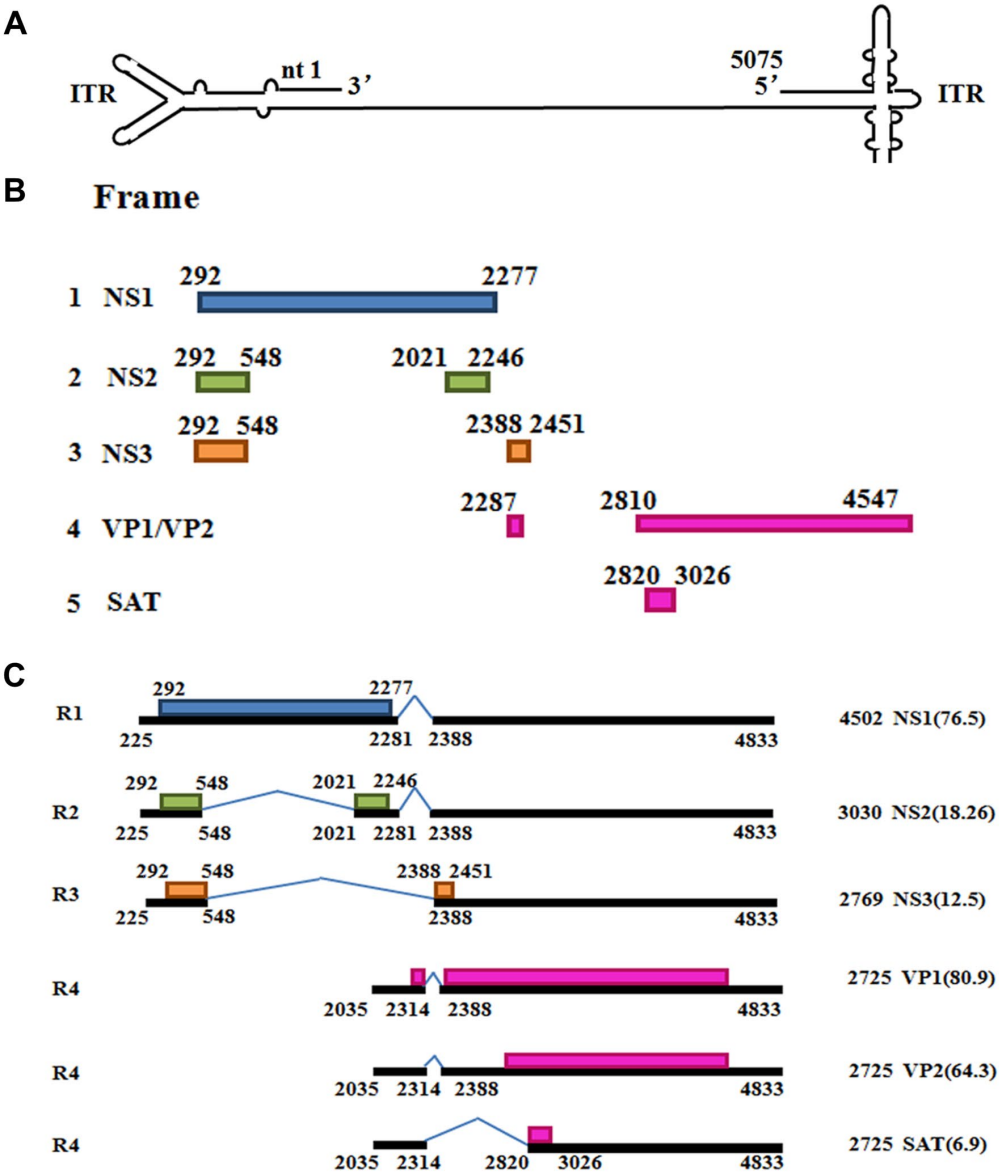
**PPV transcription map. A** PPV genome. Linear single-stranded negative PPV genome is shown. ITR, inverted terminal repeat. **B** Different open reading frames are indicated with different colors. **C** Different RNA encoding different viral proteins.

Porcine Parvovirus (PPV) is a major causative agent of stillbirth, mummification, and embryonic death in swine [1]. PPV is a single negative strand DNA virus that belongs to the species ungulate protoparvovirus 1 in the genus protoparvovirus [2], which contains two identical inverted terminal repeats (ITR) at both ends (Figure 1A). The genome contains two major open reading frames (ORF), the ORF1 encodes the nonstructural proteins NS1, NS2 and NS3, whereas the ORF2 encodes structural proteins VP1, VP2 and nonstructural protein SAT (Figures 1B, C). NS1 is a multifunctional protein, which is necessary for effective viral replication and production of infective virion [3, 4]. It is related to cell apoptosis induction [5, 6], cell cycle arrest and the suppression of type I interferon responses [7, 8]. Mature NS2 mRNA is completely located in NS1 mRNA, which is formed by NS1 mRNA after alternative splicing. During the life cycle of virus, it is very important that the ratio of NS2 to NS1 is precisely regulated. PPV NS2 protein suppresses type I interferon responses [9] and participates in viral egress from the nucleus by interacting with nuclear export factor CRM1 [10]. However, how NS1 mRNA is spliced to form NS2 mRNA is unclear.

SYNCRIP is a member of heterogeneous nuclear ribonucleoproteins (hnRNP), also known as NS1 associated protein (NSAP1) or heterogeneous nuclear ribonucleoproteins (hnRNP Q). SYNCRIP is a highly conserved cytoplasmic RNA-binding protein, which plays important roles in neuronal, myeloid leukemia stem cell and muscular development [11, 12]. Abnormal expression of SYNCRIP is associated with immune response disorders and neuro-degenerative disorders [13–15]. In addition, SYNCRIP is implicated as a key factor in the morphology and growth of the neuromuscular junction and regulation of cytoplasmic vesicle-based messenger RNA (mRNA) transport in the fly embryo [16]. SYNCRIP can interact with a lot of RNA sequence, such as UAUC [17], poly(A) [18], hEXO (GGCU/A) [19] and regulate the edition, sorting, degradation, transportation and translation of RNA [20, 21]. SYNCRIP has a similar structure to the RNA binding protein family (RBM), which contains three conserved RNA recognition motif (RRM) domains-RRM1, RRM2, and RRM3. It contains seven high-confidence RNA-bound peptides mapped to RRM 1, 2 and 3 using the RBDmap data set [19], and two high-confidence and five candidate RNA-bound peptides mapped to the amino N-terminal region of SYNCRIP [19]. The highly conserved N-terminal domain can interact with Apobec protein [22], while an irregularity, less conserved C-terminus can mediate the interaction with synaptotagmins [23]. Previous studies have found that RNA binding protein RBM38 and RBM45 regulate the expression of the 11 kDa protein of Parvovirus B19 to promote viral replication [24, 25]. SYNCRIP has been reported to be exploited by virus to promote viral replication, for example, RNA Hepatitis C virus (HCV) and mouse hepatitis virus (MHV) can bind with SYNCRIP to promote virus RNA replication [26, 27]. However, there is no report whether SYNCRIP is involved in regulating the formation of viral non-structural proteins.

In this study, we attempted to explore the mechanism of NS1 mRNA alternative splicing to produce NS2 mRNA. Using RNA-pulldown and mass spectrometry analysis, we identified and characterized a PPV NS1 mRNA-binding protein SYNCRIP. We also identified the roles of SYNCRIP in alternative splicing of NS1 mRNA to form NS2 mRNA and in modulation of NS1/NS2 ratio and PPV DNA replication, and determined the action sites of SYNCRIP on NS1 mRNA. Our proposed mechanism of SYNCRIP-mediating PPV NS1 mRNA splicing would provide potential targets for antiviral intervention and reveal a novel host function for this protein.

## Materials and methods

### Cell culture and virus

PK-15 and HEK293T cells were purchased from the American Type Culture Collection (ATCC, Manassas, VA, USA). These cells were cultured in Dulbecco modified eagle medium (DMEM) (Gebico, USA). All media were supplemented with 10% heat-inactivated fetal bovine serum (FBS; Sijiqing, Hangzhou, China) in 5% CO_2_ culture. PPV XY strain (Genbank: MK993540) was propagated in PK-15 cells. The crude PPV preparation was purified using ultracentrifugation over sucrose cushions (2 mL of 50% sucrose plus 2 mL of 20% sucrose) by Optima XPN-100 Ultracentrifugation with a SW 41 Ti rotor, at 200 000 *g* for 2 h. Virus titers in the culture supernatants were determined by the Reed-Muench method [28].

### Plasmid constructions and reactions

PPV open reading frame 1 (ORF1) coding NS1 protein and three deletion mutant fragments were constructed using the overlap PCR approach from PPV genome and cloned into the pCI-neo vector. SYNCRIP protein was constructed from PK-15 cDNA and sub-cloned into the pCDNA3.0 vector. All sequences were confirmed by sequencing analysis (Sangon Biotech, Shanghai, China). For the construction of NS2 knockout Y-PPV clone (Y-PPV^NS2−^), the three CTC in NS2 ORF of Y-PPV were mutated to TAG using the overlap PCR approach, as described previously [29].

CRISPR/Cas9 knockout cell: Lentiviral vector LentiCRISPR v2 with puro resistance gene was used to clone sgRNA sequences between *Bsm*B I sites. The sgRNA sequences were used to knockout SYNCRIP: SYNCRIP-1: 5′-CTATTGACGTCTTCATCATAGGTACC-3′; SYNCRIP-2: 5′-CACCGAGAATTCAATGAAGACGG CGCA-3′.

pLenti-NS2 and pLenti-SYNCRIP constructs: Lentiviral vector pCDH-EF1-MCS-T2A-Puro was used to clone optimized NS2 and SYNCRIP ORF between the restriction sites *Nhe* I and *Not* I. The PCR primers used in this study are shown in Table 1.

**Table 1:** **Primers used in this study**

Primers	Sequences (5′–3′)	Vectors
NS1-F	CA***CTCGAG***ATGGATTACAAGGATGACGACGATAAGGCAGCGGGAAACACTTACTC	pCI-neo
NS1-R	GGC***TCTAGA***TTATTCAAGGTTTGTTGTGGGTG	
p-NS1-F	CA***CTCGAG***ATGGCAGCGGGAAACACTTAC	pEGFP-N1
p-NS1-R	GGC***GAATTC***GCATTCAAGGTTTGTTGTGGGTG	
NS2-F	CA***CTCGAG***ATGGCAGCGGGAAACACTTAC	pEGFP-N1
NS2-R	GTC***GGATCC***GTCCAAAGCAGGCTCTTATG	
p-NS2-F	CA***GCTAGC***ATGGCAGCGGGAAACACTTAC	pCDH-EF1-MCS-T2A-Puro
p-NS2-R	GTC***GCGGCCGC***GTCCAAAGCAGGCTCTTATG	
VP1-F	CA***CTCGAG***ATGGCGCCTCCTGCAAAAAGAGCAAGAGGACTAACTCTACCAGGATAC	pEGFP-N1
VP1-R	GTC***GGATCC***GTGTATAATTTTCTTGGTATAAG	
VP2-F	CA***CTCGAG***ATGAGTGAAAATGTGGAAC	pEGFP-N1
VP2-R	GTC***GGATCC***GTGTATAATTTTCTTGGTATAAG	
SYNCRIP-F	GGC***GTCGAC***TCATGGCTACAGAACATGTTAATGGA	pGEX-4 T-1
SYNCRIP-R	GGC***GCGGCCGC***TTATTGTAACAGGTCAGGACCGG	
p-SYNCRIP-F	GGC***CTCGAG***ATGGCTACAGAACATGTTAATGGA	pCDNA3.0
p-SYNCRIP-R	GGC***TCTAGA***TTAATGGTGATGGTGATGATGGGTGGAGGCTTGTAACAGGTCAGGACCGG	

Restriction enzyme sequences are underlined.

### Antibodies and reagents

Antibodies include monoclonal mouse anti-SYNCRIP antibody (ab10687; abcam, Cambridge, MA, UK), monoclonal mouse anti-capsid primary antibody 3C9 (3C9-D11-H11; ATCC, Manassas, VA, USA), β-actin (13C000500; GenScript, Nanjing, China), Normal mouse IgG antibody (A7028; Beyotime, Shanghai, China), HRP-conjugated anti-mouse IgG (RK244131; Invitrogen, Waltham, MA, USA). T7-Biotin Labeling Transcription Kit (R11074.1; Ribo, Guangzhou, China), Dynabeads™ MyOne™ streptavidin C1 (65002; ThermoFisher, Waltham, MA, USA), Protein A PLUS-Agarose and Protein G PLUS-Agarose (SC-2002; Santa, Beijing, China).

### CRISPR-Cas9 mediated SYNCRIP knockout in PK-15 cells

Targeting sites in the SYNCRIP gene were selected using the CRISPR program (Genome Engineering, Broad Institute Cambridge, MA, USA). CRISPR-Cas9 designing and analysis methods were described in a previous study [30, 31]. In brief, two pairs of gRNA specifically targeting the porcine SYNCRIP sequence were designed using the optimized CRISPR design tool. Oligonucleotides were annealed and ligated into the Lenti-CRISPRv2 plasmid (Addgene, #52961) using the *Bsm*B I sites, respectively, and confirmed by sequencing analysis. HEK293T cells were transfected with recombinant plasmids accompanied with psPAX2 (Addgene, #12260) and pMD2.G (Addgene, #12259) to obtain recombinant lentiviruses at 72 h. Then, the culture supernatant was collected and used to infect PK-15 cells. After 48 h, the cells were selected by puromycin at a concentration of 10 μg/mL. Positive cells were obtained after about 2 weeks and then subcloned into 96-well plates for single-clone growth and saved as cell stocks.

### Reverse transcription quantitative PCR

The cDNA was synthesized using Moloney murine leukemia virus (M-MLV) kit (28025013; ThermoFisher, Waltham, MA, USA). A Real-time system (q-PCR) was used to detect PPV interrelated mRNA, β-actin as an internal control. All primer in Table 1 were synthesized (Sangon Biotech, Shanghai, China).

### Immunofluorescence assay and confocal imaging

Immunofluorescence assay was operated as previously described [32]. The cells were incubated with the primary antibodies 3C9, SYNCRIP or Cy3-NS1 mRNA Fish probe. After further washing, the cells were incubated with the secondary antibodies for 1 h at room temperature in the dark. After the final washing, the cover glasses were removed from the wells and fixed onto a glass slide, and the image was captured by a confocal microscopy.

### RNA-immunoprecipitation assay

These assays were done according to the Abcam RNA-immunoprecipitation manufacturer. Briefly, cells were harvested by trypsinization and resuspended in PBS, 2 mL freshly prepared nuclear isolation buffer was added (1.28 M sucrose, 40 mM Tris–HCl pH 7.5, 20 mM MgCl_2_, 4% Triton X-100) and the sample was kept on ice for 20 min. Then the cells were centrifuged at 2500 *g* for 15 min to obtain a pelleted nuclei. The nuclear pellet was resuspended in freshly prepared RIP buffer (150 mM KCl, 25 mM Tris pH 7.4, 5 mM EDTA, 0.5% NP40), 1 mM PMSF, 100 U/mL RNase inhibitor, as well as 1 × protease inhibitor cocktail (Sigma). Centrifugation was then used to obtain a pellet containing the nuclear membrane and debris at 13 000 *g* for 10 min. Then the specific antibody and control antibody IgG were added for 4 h at 4 ℃, then 40 μL protein G beads were added for 2 h. After centrifugation to discard the supernatant, the beads were washed three times with the RIP buffer. The RNA co-precipitated with SYNCRIP was obtained by resuspending the beads in Trizol RNA extraction reagent and further extraction, and then was analyzed by PCR; GAPDH served as a negative control.

### RNA-pulldown assay

RNA-pulldown assay was carried out as previously described [33]. Briefly, NS1 sequences were T7 transcription synthesized with biotinylation at 5′ end. 20 μL of streptavidin C1 was used for preclear nuclear extract in each sample for 30 min at 4 ℃. Then they were centrifuged at 1000 *g* for 5 min. The supernatant was collected and supplemented with yeast tRNA (0.1 μg/μL), 20 μg biotinylated NS1 mRNA or LacZ mRNA for 60 min at 4 ℃. 30 μL of streptavidin C1 was added to isolate RNA bounding to protein for 60 min at 4 ℃. Then the beads were washed five times with buffer A [150 mM KCl, 25 mM Tris pH 7.4, 5 mM EDTA, 0.5 mM DTT, 0.5% NP40, 1 mM PMSF, 100 U/mL SUPERAsin and 1 × protease inhibitor cocktail (Sigma)]. After rotating at 1000 *g* for 5 min, the precipitate was added with the protein loading buffer and loaded to precast 12% gradient Bis–Tris gel for further analysis by Coomassie brilliant blue staining. The different strips were then cut for mass spectrometry analysis.

### Electrophoretic mobility shift assay

NS1 mRNA sequence were synthesized, using a T7 in vitro transcription synthesized with biotinylation at 5′ end, following the manufacturer’s instruction. Glutathione (GST) and GST-SYNCRIP proteins were purified using the GST protein purification kit (P2262; Beyotime, China), and further incubated with biotinylation NS1 mRNA. Gel shift assays were performed using the Chemiluminescent EMSA Kit (GS009, Beyotime, China).

### Purification of GST-tagged SYNCRIP protein

Bacterially optimized SYNCRIP ORF (1–1689 bp) was cloned in pGEX-4 T-1 vector between *Sal* I and *Not* I restriction sites to express GST-SYNCRIP protein. The positive plasmids were transformed into bacteria strain Rosetta (DE3)plys. To induce fusion protein expression, isopropul β-d-1-thiogalactopyranoside (IPTG) was added to bacteria culture medium at a final concentration of 1.0 mM for 6 h at 28 ℃. GST-SYNCRIP protein was purified as previously described [34].

### Statistics

These data are shown as means ± SEM (SD) values from the three independent experiments. Statistical analyses were performed with GraphPad Prism 5 software. Immunofluorescence values were calculated using Image-Pro Plus 6.0. A value of *p* < 0.05 was considered as significant.

## Results

### PPV NS1 mRNA can specifically interact with SYNCRIP

**Figure 2:**
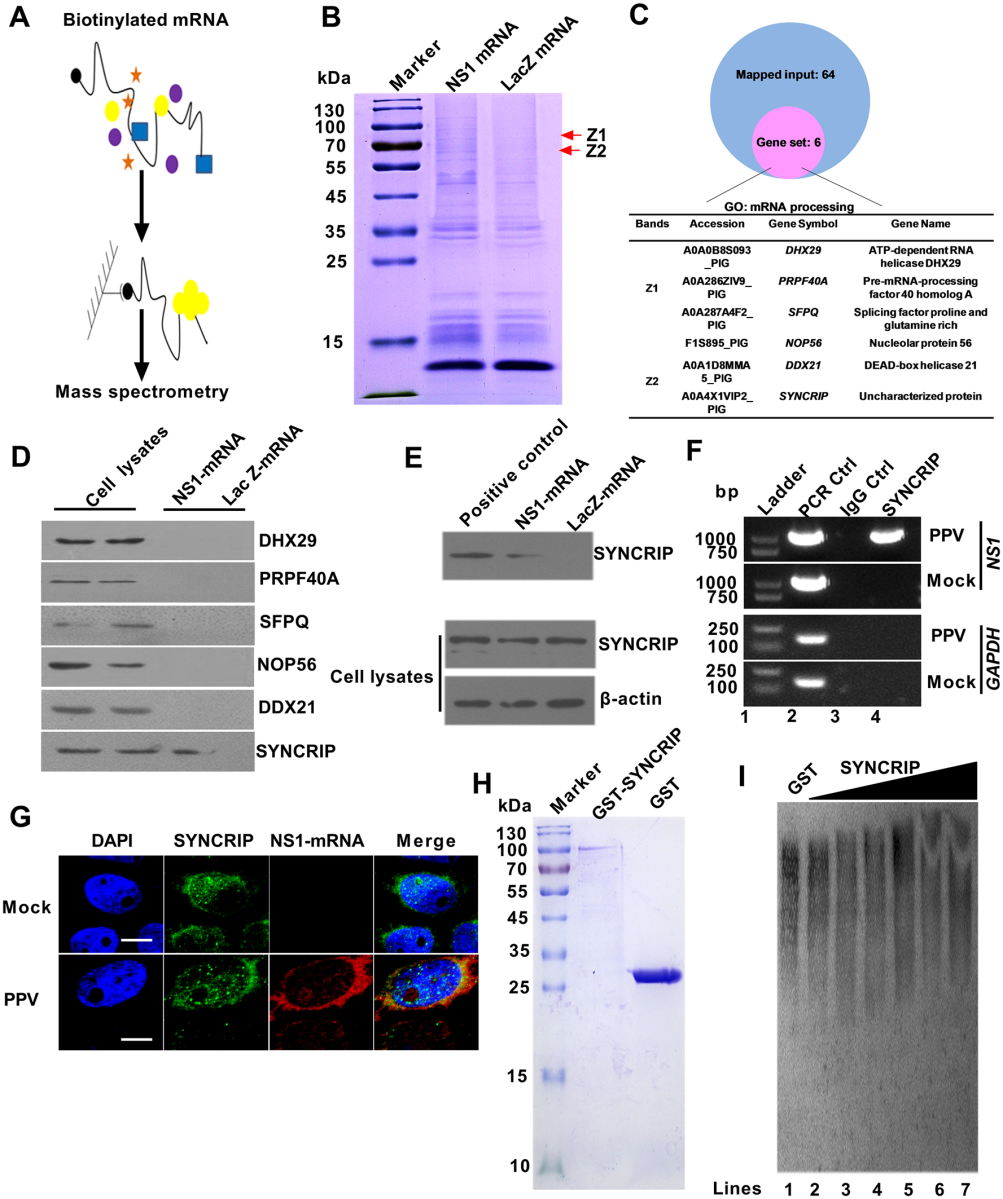
**Screening and identification of NS1 mRNA interacting host proteins. A** Experimental design for pulldown assays and identification of NS1 mRNA associated cellular proteins. NS1 mRNA and LacZ mRNA were biotinylated by transcription in vitro, and incubated with PK-15 total cell lysates. **B** Coomassie bright blue staining of biotinylated NS1 mRNA associated proteins. **C** The gene ontology (GO) analysis on NS1 mRNA interacting with host proteins. The GO analysis was performed by mRNA processing of the biological process on NS1 mRNA interacting with host proteins using webgestalt. **D** HEK293 cells were transfected with different expression vectors containing Flag tagged host proteins for 24 h. RNA-pulldown was performed with streptavidin beads, followed by western blotting using the anti-Flag antibodies. **E** Western blotting identified the interaction between NS1 mRNA and endogenous SYNCRIP protein in PPV-infected PK-15 cells by RNA-pulldown. **F** RNA-immunoprecipitation identified the interaction between NS1 mRNA and endogenous SYNCRIP protein in PPV-infected PK-15 cells. An anti-SYNCRIP antibody or negative control IgG was used to pull down RNA–protein complexes. Recovered cDNA from PPV-infected PK-15 cells was examined for viral RNA by PCR with primer sets of *NS1* and *GAPDH*, Y-PPV plasmid was used as a template for positive controls of PCR. **G** Laser confocal identify the interaction between NS1 mRNA and endogenous SYNCRIP protein in PPV-infected PK-15 cells. **H** Identification of GST and GST-SYNCRIP protein purification. **(I)** EMSA identified the interaction between NS1 mRNA and endogenous SYNCRIP protein. Scale bar = 10 μm.

Since NS2 mRNA is completely contained in NS1 mRNA, to determine how PPV NS1 mRNA regulate NS2 expression through alternative splicing, we sought to identify intracellular NS1 mRNA binding factors using an unbiased approach. Full-length NS1 mRNA was transcribed with biotinylated nucleotides in vitro. Used partial LacZ mRNA without protein-coding potential served as a negative control. Biotinylated NS1 mRNA or LacZ mRNA were incubated with total protein extracted from PK-15 cells and pulled down with streptavidin (Figure 2A). The associated proteins were analyzed by SDS-PAGE and Coomassie blue staining. Two different strips specifically presented in the NS1 mRNA pull-down not LacZ mRNA samples were excised and analyzed by mass spectrometry (Figure 2B), which identified sixty-four potential binding proteins (Additional file 1), six of them were identified to be involved in mRNA processing using gene ontology (Figure 2C).

To determine potential proteins that specifically interact with NS1 mRNA, first, we used online software catRAPID to predict the interaction between NS1 mRNA and the six host proteins. The results show that only SYNCRIP could interact with NS1 mRNA (Data not shown). In agreement with that, RNA-pulldown assay shows that only SYNCRIP could interact with NS1 mRNA in HEK293 cells, but not DHX29, PRPF40A, SFPQ, NOP56, DDX21 (Figure 2D). In addition, biotinylated NS1 mRNA was also confirmed to interact with the endogenous SYNCRIP protein in PK-15 cells (Figure 2E). To substantiate this interaction, anti-SYNCRIP antibody was used to immunoprecipitate endogenous SYNCRIP and its binding RNA from the nuclear extracts of PPV-infected PK-15 cells, and RNA interacting with SYNCRIP were collected and analyzed. We detected the enrichment of NS1 mRNA, but not control GAPDH RNA, in the immunoprecipitates of anti-SYNCRIP compared with the IgG control (Figure 2F). Furthermore, fluorescence in situ hybridization assay showed that SYNCRIP and NS1 mRNA co-localized in PPV-infected PK-15 cells (Figure 2G). Meanwhile, purified GST-tagged SYNCRIP directly binded with NS1 mRNA in vitro (Figure 2H). Electrophoretic mobility shift assay shows that shift speed of biotinylated NS1 mRNA was slowed down with the increase of GST-SYNCRIP concentration (Figure 2I lines 3–6), suggesting that SYNCRIP can specifically interact with NS1 mRNA.

### PPV infection up-regulates SYNCRIP expression in vitro and in vivo

**Figure 3:**
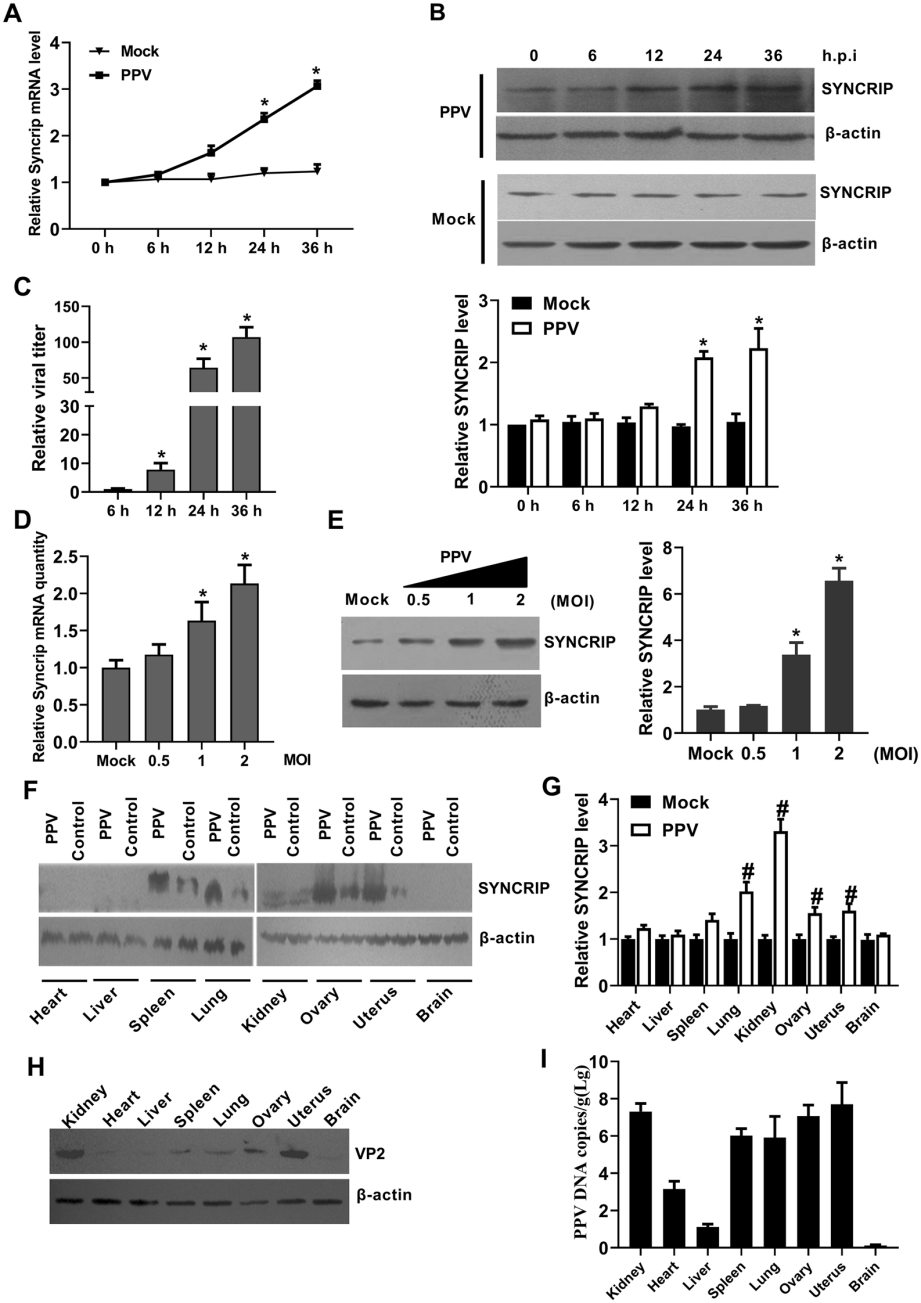
**PPV infection up-regulates SYNCRIP expression in vitro and in vivo. **PK-15 cells were infected with 1 MOI of PPV or mock infection for the time indicated, the mRNA level of SYNCRIP (**A**), the protein level of SYNCRIP (**B**) and the relative viral titers of different infection times were measured by TCID_50_ (**C**). **D-E** qPCR and western blotting analysis of SYNCRIP mRNA and protein levels in PK-15 cells infected with different doses of PPV (MOI = 0, 0.5, 1, or 2) for 24 h. β-actin served as an internal control. **F–G** Western blotting and qPCR analysis of SYNCRIP protein and mRNA levels in different tissues of PPV infected pregnant sows. **H–I** Western blotting and q-PCR analysis of viral VP2 protein levels and DNA copies in different tissues of PPV infected pregnant sows. β-actin served as an internal control. The results are shown as the mean ± SD (*n* = 3). *p < 0.05, versus mock infected cells at same time points. ^#^*p* < 0.05, versus mock tissues at same tissue.

To determine the roles of porcine SYNCRIP in the process of PPV infection, we further investigated the expression profiles of SYNCRIP in PPV-infected PK-15 cells. Time-course experiments show that SYNCRIP mRNA significantly increased at 12 h post-infection (hpi) and reached the highest levels at 36 hpi (Figure 3A). Consistent with the changes of SYNCRIP mRNA levels, the SYNCRIP protein gradually increased at 12 hpi and peaked at 36 hpi (Figure 3B). In contrast with the pattern of SYNCRIP, the PPV viral titer gradually increased during the 12 to 36 hpi (Figure 3C).

To further confirm the effects on the expression of SYNCRIP during PPV infection, PK-15 cells were infected with different doses of PPV (MOI = 0, 0.5, 1, or 4) for 24 h. The results show that SYNCRIP expression significantly increased with the doses of PPV infection, and the expression level of SYNCRIP is associated with viral infection level (Figures 3D, E). These results demonstrate that the expression of SYNCRIP is upregulated by PPV infection in vitro. To further confirm whether the expression of SYNCRIP is up-regulated by PPV infection in vivo*,* we examined the mRNA and protein levels of SYNCRIP in different tissues of pregnant sows infected by PPV. The results show that the mRNA level of SYNCRIP in major targeted tissues (spleen, lung, kidney, ovary and uterus) increased compared to those in the nonpathological tissues (heart, liver and brain) of PPV-infected pregnant sows at 35 days post-infection (dpi) (Figure 3F). Consistently, SYNCRIP protein levels varied in different tissues of control pregnant sows and also apparently increased in lung, kidney, ovary and uterus of PPV-infected pregnant sows at 35 days post-infection (Figure 3G). Simultaneously, PPV VP2 protein levels and viral loads were higher in major pathological tissues (spleen, lung, kidney, ovary and uterus) than those in the nonpathological tissues (heart, liver and brain) of PPV-infected pregnant sows (Figures 3H, I). These results demonstrate that the expression of SYNCRIP was regulated in PPV-infected cells and tissues.

### CRISPR-Cas9 mediated knockout of SYNCRIP impairs PPV replication

**Figure 4:**
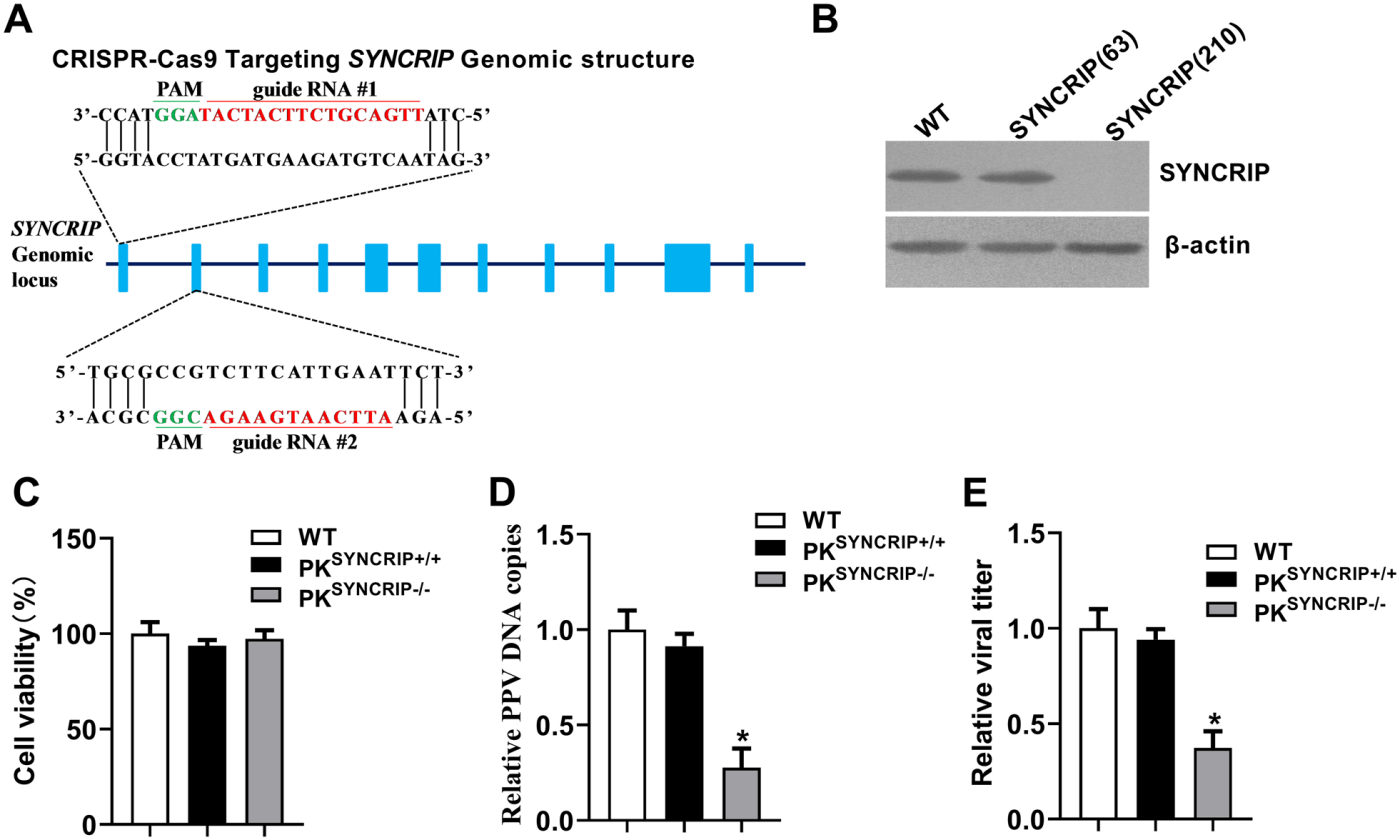
**Knockout of SYNCRIP inhibits the viral replication in PPV‑infected cells. A** Schematic chromatogram representation of sgRNA targeting at the SYNCRIP genomic region. PAM sequences are underlined and highlighted in green. sgRNA targeting sites are underlined and highlighted in red. Red arrows indicate gRNA targeting sites. **B** Western blotting analysis of the SYNCRIP expression in PK-15 cells infected with CRISPR/Cas9 lentivirus and then selected by puromycin. The lentiviruses contain gRNA-63 and gRNA-210. **C** Cell viability of cell lines stably knockout for SYNCRIP. **D-E** Knockout of SYNCRIP inhibits PPV progeny virion production. PK-15, PK^SYNCRIP+/+^ and PK^SYNCRIP−/−^ cells were infected with PPV (MOI = 1) for 24 h. The relative fold-change PPV DNA copies and viral titers were determined by q-PCR and TCID_50_ assay. The results are shown as the mean ± SD (*n* = 3). *p < 0.05 versus PK-15 cells with the same treatment.

To determine the roles of SYNCRIP in the process of PPV infection, we used CRISPR/Cas9 genomic editing system to construct PK-15 cells with SYNCRIP gene deletion. Two RNA guides (gRNA-63 and gRNA-210) were designed to target two sites of the exon 1 and exon 2 of SYNCRIP genome sequences respectively (Figure 4A). Single cell clones SYNCRIP (63) and SYNCRIP (210) were selected from cells infected with Cas9 recombinant lentivirus encoding gRNA-63 or gRNA-210 respectively. Western blotting analysis shows that SYNCRIP expression was deficient in the SYNCRIP (210) cells clone, but was not affected in the SYNCRIP (63) cell clone (Figure 4B). We named these two cells as PK^SYNCRIP−/−^ and PK^SYNCRIP+/+^ cells, respectively. The cell viability assay shows that the viability of PK^SYNCRIP+/+^ and PK^SYNCRIP−/−^ cells were not different from PK-15 cells (wild-type) (Figure 4C). Next, PK-15, PK^SYNCRIP+/+^ and PK^SYNCRIP−/−^ cells were infected with equal amounts of PPV (MOI = 1) for 24 hpi. PPV DNA copies and viral titers significantly decreased in PK^SYNCRIP−/−^ cells compared with PK-15 and PK^SYNCRIP+/+^ at 24 hpi (Figures 4D, E). These results suggest that SYNCRIP deficiency significantly impaired PPV replication.

### Over-expression of SYNCRIP leads to the reduction of NS1 mRNA and protein expression

**Figure 5:**
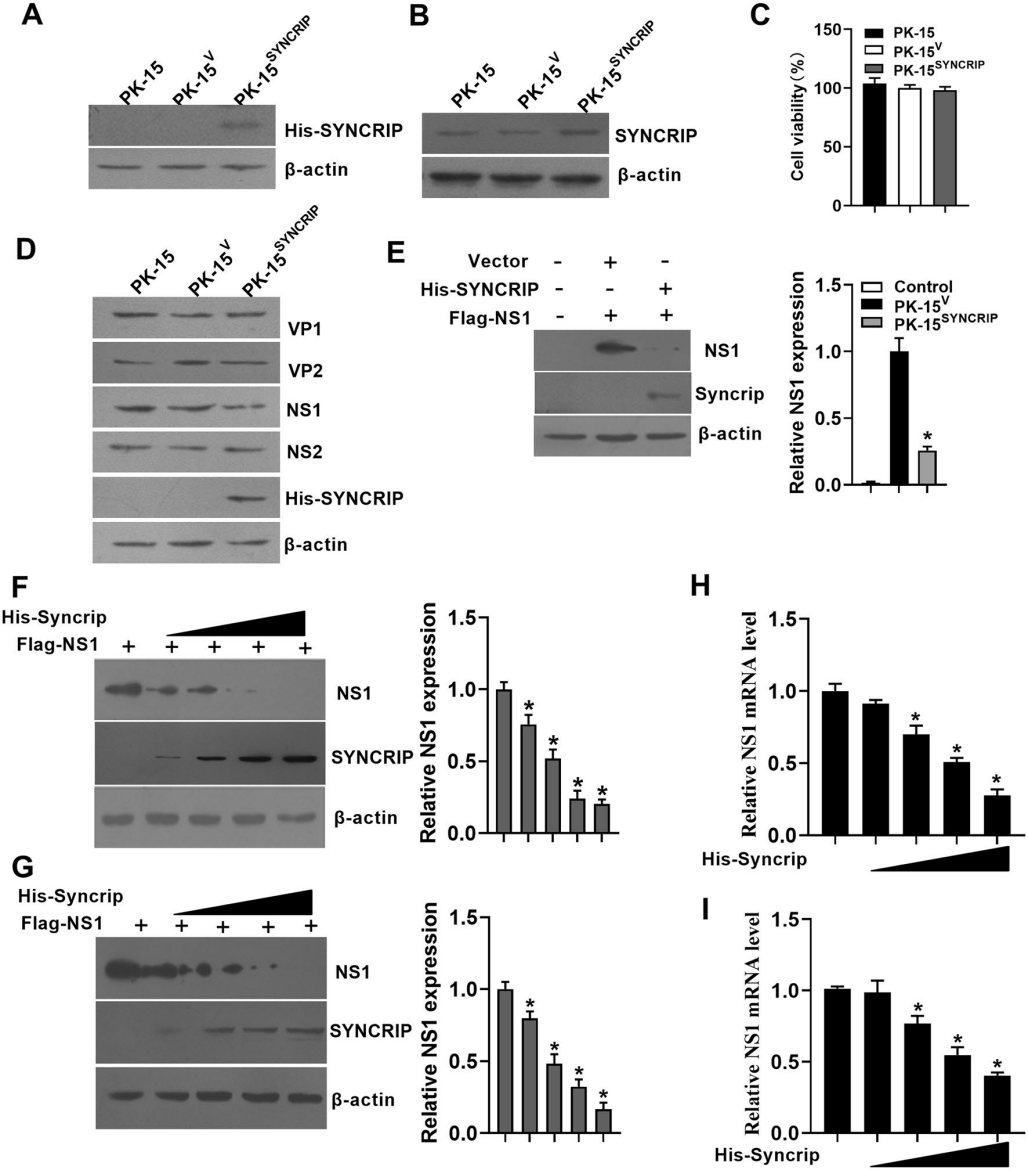
**Over-expression of SYNCRIP leads to the reduction of NS1 mRNA and protein expression. A** Western blotting analysis of SYNCRIP protein levels in PK-15 cells with SYNCRIP over-expression using anti-His monoclonal antibodies. PK-15 as the cells without transfection, PK^V^ as the cells transfected with blank vector, PK^SYNCRIP^ as the cells transfected with the vector expressing His-SYNCRIP. **B** Western blotting analysis of SYNCRIP protein levels in PK-15 cells with SYNCRIP over-expression using SYNCRIP monoclonal antibodies. **C** Cell viability of cell lines stably overexpressing SYNCRIP. **D** Western blotting analysis of viral NS1, NS2, VP1 and VP2 proteins expression levels in PK-15 cells with SYNCRIP over-expression. **E** Western blotting analysis of NS1 protein expression levels in PK-15 cells of SYNCRIP over-expression. **F** Western blotting analysis of NS1 protein levels in PK-15 cells co-transfected with different doses of SYNCRIP (0, 1, 2, 4 or 8 μg) for 24 h.**G** Western blotting analysis of NS1 protein levels in ST cells co-transfected with different doses of SYNCRIP (0, 1, 2, 4 or 8 μg) for 24 h. **H** Q-PCR analysis of NS1 mRNA levels in PK-15 cells co-transfected with different doses of SYNCRIP (0, 1, 2, 4 or 8 μg) for 24 h. **I** Q-PCR analysis of NS1 mRNA levels in ST cells co-transfected with different doses of SYNCRIP (0, 1, 2, 4 or 8 μg) for 24 h. The results are shown as the mean ± SD (*n* = 3). *p < 0.05 versus untreated cells with the vector pCDNA-His-SYNCRIP over-expression.

To detect whether the interaction between SYNCRIP and NS1 mRNA will affect the expression of NS1, we established a cell line stably expressing pCDNA-His-SYNCRIP, named as PK-15^SYNCRIP^ (Figures 5A, B). The cell viability assay shows that the viability of overexpressing SYNCRIP cells was not significantly different from PK-15 or PK-15^V^ (Figure 5C). When recombinant plasmids (pEGFP-NS1, pEGFP-NS2, pEGFP-VP1 and pEGFP-VP2) expressing NS1, NS2, VP1, or VP2 were transfected into PK-15^SYNCRIP^, PK-15 or PK-15^V^ cells, the level of NS1 protein decreased in PK-15^SYNCRIP^ cells, while the protein level of VP1, VP2 or NS2 were not changed, compared to those in PK-15 or PK-15^V^ control cells (Figure 5D). We further tested whether transient overexpression of SYNCRIP could also decrease NS1 levels, and found that SYNCRIP overexpression did decrease the amount of NS1 protein in PK-15 cells (Figure 5E). This was consistent with the results that the expression of NS1 decreased gradually in PK-15 cells or ST cells, with the increase of the expression of SYNCRIP plasmid (Figures 5F, G). Consistently, we noted that NS1 mRNA was significantly decreased in the cells that overexpressed SYNCRIP compared to the cells without overexpression (Figures 5H, I), suggesting that SYNCRIP could regulate NS1 expression at the mRNA and protein levels.

### SYNCRIP regulates the ratio of NS1 mRNA to NS2 mRNA and the replication of PPV

**Figure 6:**
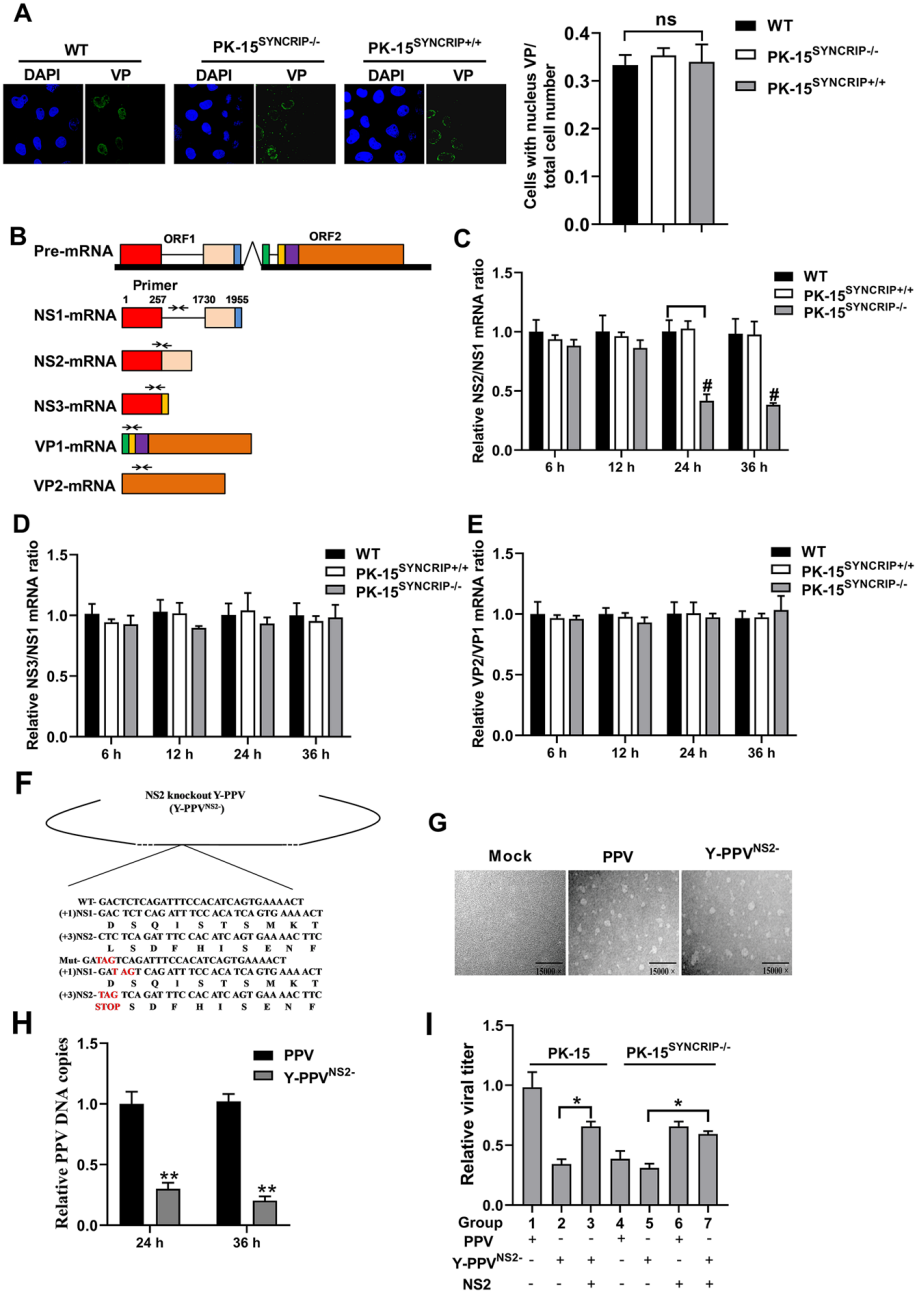
**SYNCRIP regulates the ratio of NS1 mRNA to NS2 mRNA and the effect on viral replication. A** Laser confocal identification of the effect of SYNCRIP on viral entry in the presence of the translation inhibitor cycloheximide. **B** NS1 mRNA and its alternatively spliced products are depicted and the arrowheads show primer positions for detection of various mRNA in the following experiments. Cells were infected with PPV, and total RNA was isolated and analyzed by q-PCR with primers specific to NS1, NS2, NS3, VP1 and VP2 mRNA. Ratios of NS2 mRNA to NS1 (**C**) or to NS3 (**D**), or VP2 to VP1 (**E**) at the indicated times are presented. **F** Diagram of NS2 knockout Y-PPV^NS2−^. The NS2 protein knockout Y-PPV clone (Y-PPV^NS2−^) is diagrammed and shown with mutations of three translation initiation codons from CTC to TAG. **G** Transmission electron microscopy of rescued Y-PPV^NS2−^ virus. Viral particle morphology was observed using transmission electron microscopy. **H** Q-PCR analysis of parental PPV and Y-PPV^NS2−^ relative DNA copies at different infection time points. **I** SYNCRIP knockout decreases viral DNA replication via reduction of NS2 protein expression. PK-15 cells were infected with Y-PPV or Y-PPV^NS2−^, and NS2 proteins were in part of these cells as indicated. PK-15^SYNCRIP−/−^ cells or NS2 protein expressing PK-15^SYNCRIP−/−^ cells were infected with Y-PPV. At 24 h post-infection, the supernatants from each infected sample was collected and used for virion quantification by TCID_50_. The results are shown as the mean ± SD (*n* = 3). ns: no significant difference; ^#^*p* < 0.05 versus PK-15 cells infected with PPV at the same time points. **p < 0.01 versus PK-15 cells with the same treatment.

To investigate the function of SYNCRIP in the PPV life cycle, we first assessed whether SYNCRIP depletion affected viral entry by examining the localization of viral capsid protein (VP) in SYNCRIP-depleted cells after PPV infection in the presence of the translation inhibitor cycloheximide. Compared to the control, SYNCRIP knockout did not decrease nuclear VP staining at 2 hpi (Figure 6A), indicating that SYNCRIP deficiency did not affect PPV entry. To investigate the potential effect on the splice of mRNA, the mRNA levels of NS2 and NS1 were measured by qPCR as shown in Figure 6B. Indeed, the ratio of NS2 to NS1 mRNA decreased in PPV-infected SYNCRIP depleted cells (Figure 6C). As NS2 mRNA, NS3 mRNA was also derived from the alternative splicing of NS1 mRNA segment, but we did not find a significant difference in the ratio of NS3 to NS1 mRNA among PK-15, PK^SYNCRIP+/+^ and PK^SYNCRIP−/−^ cells (Figure 6D). However, for another viral mRNA segment, VP mRNA, which could generate VP1 and VP2 mRNA through alternative splicing, no differences were observed between the control and SYNCRIP depleted cells in the ratio of VP2 to VP1 mRNA (Figure 6E). Taken together, these findings revealed that SYNCRIP could regulate the production of NS2 mRNA through alternative splicing of NS1 mRNA.

To further determine the role of NS2 in PPV replication, we used PPV infectious clone Y-PPV as the operating platform to construct the NS2 mutant strain using the overlap technique for site-directed mutagenesis (Figure 6F), and further observed whether NS2 mutant could package into virion by transmission electron microscopy. The results show that the NS2 mutant could package into virion (Figure 6G), but the NS2 mutant replication rate significantly decreased compared to parental PPV (Figure 6H). To confirm whether SYNCRIP knockout decreased viral DNA replication via inhibition of the NS2 protein, we tested the relative viral titer of PPV or Y-PPV^NS2−^ in PK-15 cells and SYNCRIP deficient cells depending on whether they express NS2 or not via infection with NS2 protein expressing lentivirus. The results show that NS2 protein complementation significantly increased PPV production in PK-15 cells infected by Y-PPV^NS2−^ (Figure 6I, compare lane 2 and 3). Similarly, NS2 protein complementation also significantly increased PPV production in Y-PPV^NS2^-infected PK-15^SYNCRIP−/−^ cells (Figure 6I, compare lane 5 and 7). Meanwhile, we noted that PPV replication levels were reduced in Y-PPV^NS2−^ infected PK-15 cells, PPV or Y-PPV^NS2^-infected PK-15^SYNCRIP−/−^ cells, but did not show a significant difference among these three cells (Figure 6I, compare lane 2, 4 and 5). Similarly, PPV replication levels did not show a significant difference among Y-PPV^NS2^-infected PK-15 cells, PPV or Y-PPV^NS2^-infected PK-15^SYNCRIP−/−^ cells when these cells exogenously expressed NS2 protein (Figure 6I, compare lane 3, 6 and 7). However, PPV replication levels were higher in Y-PPV^NS2^-infected PK-15 cells, PPV or Y-PPV^NS2^-infected PK-15^SYNCRIP−/−^ cells when these cells exogenously expressed NS2 protein as compared to those in these cells without NS2 exogenous expression (Figure 6I). Taken together, these results confirm that SYNCRIP plays an important role in PPV replication via regulation of NS2 protein expression.

### SYNCRIP affects the NS1 expression by acting on the 3′-terminal sites of NS2 mRNA

**Figure 7:**
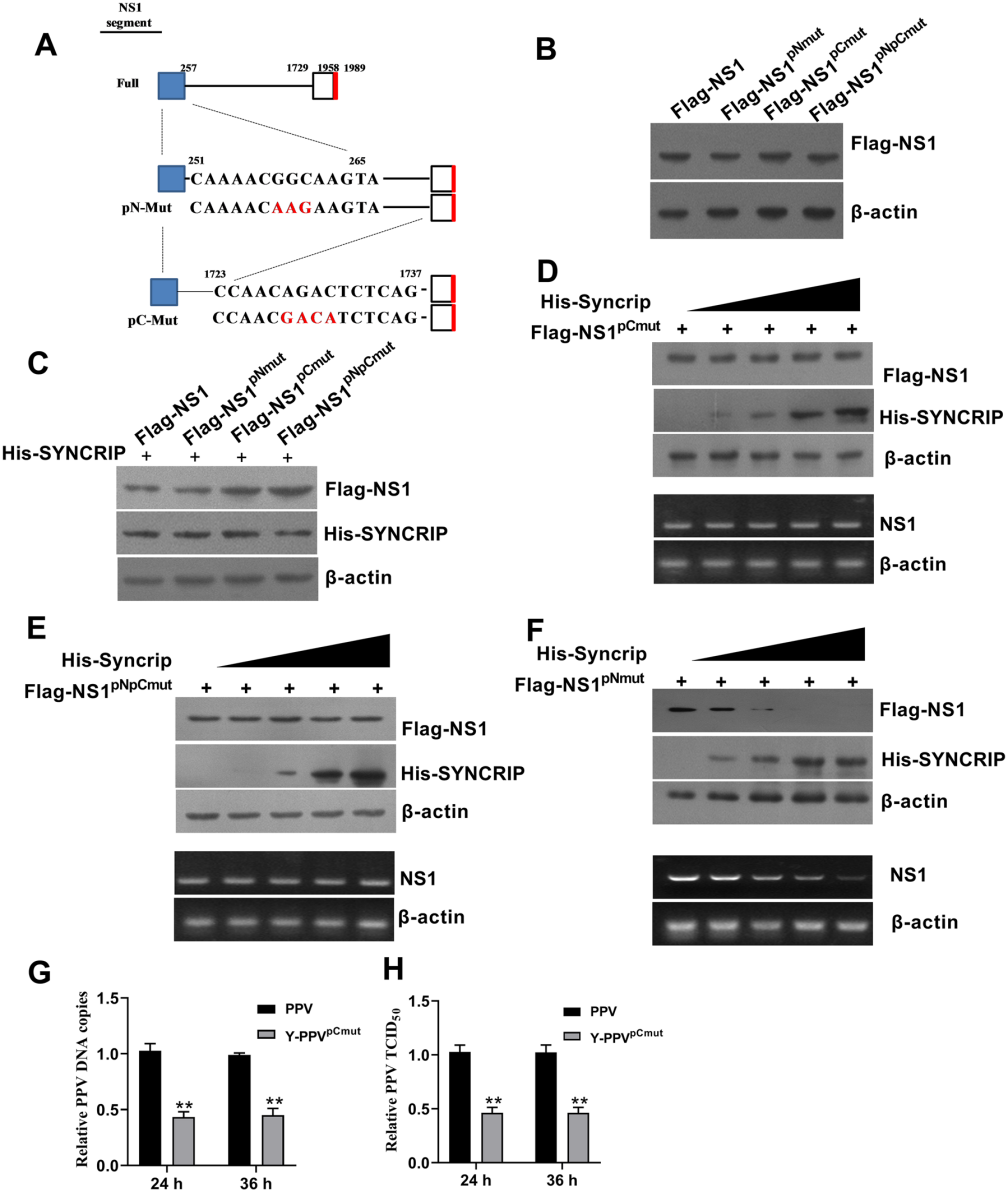
**SYNCRIP affects NS1 expression by acting on the 3′-terminal site of NS2 mRNA. A** A schematic diagram of NS1 mRNA mutation site. **B** Western blotting analysis of the expression of different NS1 mutants in PK-15^SYNCRIP−/−^ cells. **C** Western blotting analysis of viral parental NS1, NS1^pNmut^, NS1^pCmut^ and NS1^pNpCmut^ protein expression levels in PK-15 cells with SYNCRIP over-expression. **D** Western blotting analysis of NS1^pCmut^ protein levels (upper) and mRNA levels (lower) in PK-15 cells co-transfected with different doses of SYNCRIP for 24 h. **E** Western blotting analysis of NS1^pNpCmut^ protein levels (upper) and mRNA levels (lower) in PK-15 cells co-transfected with different doses of SYNCRIP for 24 h. **F** Western blotting analysis of NS1^pNmut^ protein levels (upper) and mRNA levels (lower) in PK-15 cells co-transfected with different doses of SYNCRIP for 24 h. **G** The effects of NS1^pCmut^ mutation on PPV DNA copy number were determined by q-PCR assay. (H) The effects of NS1^pCmut^ mutation on PPV viral titer were determined by TCID_50_ assay. The results are shown as the mean ± SD (*n* = 3). **p < 0.01 versus PK-15 cells with the same treatment at the same time points.

To further identify how SYNCRIP regulates NS2 mRNA splicing, we constructed different NS1 5′-terminal and 3′-terminal splicing site mutants in NS1 mRNA (Figure 7A), and detected and compared the levels of NS1 protein when cells were transfected with the different mutant constructs together with pCDNA-His-SYNCRIP or without. The results show that the expression of NS1 did not show a difference among these cells transfected with Flag-NS1, Flag-NS1^pNmut^, Flag-NS1^pCmut^ or Flag-NS1^pNpCmut^ when these cells lacked SYNCRIP expression (Figure 7B). However, when cells were expressed with SYNCRIP, the expression of NS1 significantly reduced in the cells transfected with Flag-NS1 or Flag-NS1^pNmut^ compared to the cells transfected with Flag-NS1^pCmut^ and Flag-NS1^pNpCmut^ (Figure 7C). With the increase of SYNCRIP expression, the expression of Flag-NS1^pCmut^ and Flag-NS1^pNpCmut^ did not gradually decrease in cells, but Flag-NS1 and Flag-NS1^pNmut^ gradually decreased in cells (Figures 7D–F and Figure 5F). Consistently, we noted that NS1 mRNA did not decrease in the cells transfected with Flag-NS1^pCmut^ or Flag-NS1^pNpCmut^ with the increase of SYNCRIP, but decreased in the cells transfected with Flag-NS1^pNmut^ when SYNCRIP expression gradually increased (Figures 7D–F), suggesting that SYNCRIP targeted the 3′-terminal site of NS1 mRNA to regulate the production of NS2 mRNA. To further detect the effects of the 3′-terminal mutation site of NS1 mRNA on PPV replication, we constructed a 3′-terminal site mutant of NS1 infectious clone (Y-PPV^pCmut^) using PPV infectious clone Y-PPV by site-directed mutagenesis. The results show that the replication rate of Y-PPV^pCmut^ mutants significantly decreased compared to parental PPV (Figures 7G, H).

## Discussion

PPV is a major causative agent of stillbirth, mummification, and embryonic death in swine [1]. The PPV genome encodes four nonstructural proteins NS1, NS2, NS3 and late SAT protein [35, 36]. NS1 protein has helicase and nickase activities [37, 38], and can induce apoptosis and cell cycle arrest [39]. NS2 protein can block the expression of IFN-β induced by ds-RNA [9], and associates with the nuclear egress of progeny virions efficiently [40]. A late non-structural protein SAT is expressed from the same mRNA as VP2, which can contribute to viral spreading by inducing irreversible endoplasmic reticulum stress in the progress of PPV infection [2].

Alternative splicing process of Parvovirus pre-mRNA plays a key role in regulating the expression of viral proteins [25, 41]. Mature PPV NS2 mRNA is completely located in NS1 mRNA, which is formed by alternative splicing of NS1 mRNA. SYNCRIP has been reported as a splicing factor to be involved in post-transcriptional [42, 43], miRNA exosomal sorting process [21], synaptic protein mRNA expression [11] and RNA virus replication [26, 27]. SYNCRIP, a member of heterogeneous nuclear ribonucleoproteins (hnRNP), is highly conserved among different species. SYNCRIP, which is a cytoplasm RNA-binding protein contains three typical domains, RRM1 domain, RRM2 domain and RRM3 domain. Porcine SYNCRIP is encoded by the porcine SYNCRIP gene and possesses 99.84% identity to human SYNCRIP (GenBank: NM006372). In this study, we first reported that NS1 mRNA could directly interact with SYNCRIP, and found that SYNCRIP could be detected in most tissues of pigs, such as the spleen, lung, kidney, ovary and uterus. The expression of porcine SYNCRIP was upregulated with PPV infection levels. Importantly, we found that SYNCRIP knockout significantly impaired PPV replication. This result agrees with the previous observations that SYNCRIP is involved in MHV and HCV RNA replication [26, 27].

Previous studies have reported that some RNA binding proteins are involved in alternative splicing either viral RNA or host cell RNA. HnRNP K can interact with influenza virulence factor NS1 binding protein (NS1-BP) to promote the splicing of the viral M1 RNA into M2 RNA [44], also modulate CD44 alternative splicing during the epithelial mesenchymal transition [45], and regulate the neuronal differentiation factor TRF2 alternative splicing. RBM38 is an essential host factor of B19V pre-mRNA splicing and it is important for the expression of the non-structural 11 kDa protein [24]. Because the hnRNP family has a highly conserved RNA recognition region, we speculated that SYNCRIP may directly interact with PPV virulence factor NS1 mRNA and regulate NS1 expression. When recombinant plasmids (pEGFP-NS1, pEGFP-NS2, pEGFP-VP1 and pEGFP-VP2) were transfected into PK-15^SYNCRIP^, PK-15 and PK-15^V^ cells, the level of NS1 protein and mRNA were decreased in PK-15^SYNCRIP^ cells, compared to that in PK-15 or PK-15^V^ control cells, but the levels of VP1 and VP2 were not decreased in PK-15^SYNCRIP^ cells, compared to those in PK-15 or PK-15^V^ control cells. In addition, we noted that NS1 mRNA was significantly decreased in the PPV-infected PK-15^SYNCRIP^ cells relative to PK-15 and PK-15^V^ cells. Furthermore, NS2 mutant’s replication rate significantly decreased compared to parental PPV. These results suggest that SYNCRIP targets NS1 mRNA to affect the production of NS2-mRNA and regulated replication of PPV during PPV infection.

To further identify how SYNCRIP regulates NS1 mRNA splicing, we constructed different NS1 3′-terminal and 5′-terminal splicing site mutants in NS1 mRNA. The results show that the expression of NS1 in the Flag-NS1 and Flag-NS1^pNmut^ were significantly reduced by SYNCRIP co-expression, while the expression of NS1 in Flag-NS1^pCmut^ and Flag-NS1^pNpCmut^ were not decreased by SYNCRIP co-expression. The increasing of SYNCRIP expression also did gradually decrease the expression of Flag-NS1 and Flag-NS1^pNmut^, but did not affect the expression of Flag-NS1^pCmut^ and Flag-NS1^pNpCmut^. Consistently, we noted that NS1 mRNA was obviously decreased in the cells that transfected with Flag-NS1 and Flag-NS1^pNmut^ expressing vectors, but not in the cells that were transfected with Flag-NS1^pCmut^ and Flag-NS1^pNpCmut^ expressing vectors. When these cells overexpressed SYNCRIP, these results demonstrate that SYNCRIP targets the 3′-terminal of NS1 mRNA to regulate the production of NS2 mRNA. Meanwhile, a 3′-terminal site mutant of NS2 infectious clone (Y-PPV^pCmut^) shows a lower replication rate compared to parental PPV, further demonstrating that the 3′-terminal of NS1 mRNA as the splicing target of SYNCRIP is important for NS2 expression and PPV replication.

In summary, the data presented here demonstrate that a porcine RNA binding protein SYNCRIP can directly interact with PPV NS1 mRNA to modulate PPV replication by targeting the 3′-termial site of NS1 mRNA to regulate NS2 expression. This finding will provide one possible antiviral target for porcine parvovirus disease.

## Supplementary Information

**Additional file 1. Mass spectrometry data.**

## Data Availability

All data generated or analyzed during this study are included in this published article.
