# Supplementary material for: SYNCRIP facilitates porcine parvovirus viral DNA replication through the alternative splicing of NS1 mRNA to promote NS2 mRNA formation
Source: Vet Res. 2021 May 25;52:73. doi: 10.1186/s13567-021-00938-6 (PMC8152309; doi:10.1186/s13567-021-00938-6)
Supplement: Supplementary file 1 — Additional file 1. Mass spectrometry data. [file 13567_2021_938_MOESM1_ESM.doc]

**Additional file 1 Mass** spectrometry data

| 1 | [A0A287BHY5_PIG](http://sale-depta-pc/mascot/cgi/master_results_2.pl?file=20190124%2FF003161.dat;pr.eh=1%2C1p;pr.page=1;pr.per_page=1;pr.show=proteins) | Keratin, type II cytoskeletal 2 epidermal OS=Sus scrofa OX=9823 GN=KRT2 PE=1 SV=1 |
| --- | --- | --- |
| 2 | [A0A286ZT13_PIG](http://sale-depta-pc/mascot/cgi/master_results_2.pl?file=20190124%2FF003161.dat;pr.eh=2%2C2p;pr.page=2;pr.per_page=1;pr.show=proteins) | Serum albumin OS=Sus scrofa OX=9823 GN=ALB PE=1 SV=1 |
| 3 | [A0A0B8RVJ1_PIG](http://sale-depta-pc/mascot/cgi/master_results_2.pl?file=20190124%2FF003161.dat;pr.eh=3%2C3p;pr.page=3;pr.per_page=1;pr.show=proteins) | Ribosome binding protein 1 OS=Sus scrofa domesticus OX=9825 GN=RRBP1 PE=4 SV=1 |
| 4 | [F1RS45_PIG](http://sale-depta-pc/mascot/cgi/master_results_2.pl?file=20190124%2FF003161.dat;pr.eh=4%2C4p;pr.page=4;pr.per_page=1;pr.show=proteins) | DNA topoisomerase 2 OS=Sus scrofa OX=9823 GN=TOP2B PE=1 SV=3 |
| 5 | [LMNA_PIG](http://sale-depta-pc/mascot/cgi/master_results_2.pl?file=20190124%2FF003161.dat;pr.eh=5%2C5p;pr.page=5;pr.per_page=1;pr.show=proteins) | Prelamin-A/C OS=Sus scrofa OX=9823 GN=LMNA PE=3 SV=1 |
| 6 | [A0A287A4F2_PIG](http://sale-depta-pc/mascot/cgi/master_results_2.pl?file=20190124%2FF003161.dat;pr.eh=6%2C6p;pr.page=6;pr.per_page=1;pr.show=proteins) | Splicing factor proline and glutamine rich OS=Sus scrofa OX=9823 GN=SFPQ PE=4 SV=1 |
| 7 | [A0A287BSL5_PIG](http://sale-depta-pc/mascot/cgi/master_results_2.pl?file=20190124%2FF003161.dat;pr.eh=7%2C7p;pr.page=7;pr.per_page=1;pr.show=proteins) | Kinectin OS=Sus scrofa OX=9823 GN=KTN1 PE=1 SV=1 |
| 8 | [I3LDM6_PIG](http://sale-depta-pc/mascot/cgi/master_results_2.pl?file=20190124%2FF003161.dat;pr.eh=8%2C8p;pr.page=8;pr.per_page=1;pr.show=proteins) | Keratin 3 OS=Sus scrofa OX=9823 GN=KRT3 PE=1 SV=2 |
| 9 | [A0A286ZHV7_PIG](http://sale-depta-pc/mascot/cgi/master_results_2.pl?file=20190124%2FF003161.dat;pr.eh=9%2C9p;pr.page=9;pr.per_page=1;pr.show=proteins) | Heparan sulfate proteoglycan 2 OS=Sus scrofa OX=9823 GN=HSPG2 PE=1 SV=1 |
| 10 | [A0A287A7R5_PIG](http://sale-depta-pc/mascot/cgi/master_results_2.pl?file=20190124%2FF003161.dat;pr.eh=10%2C10p;pr.page=10;pr.per_page=1;pr.show=proteins) | IQ motif containing GTPase activating protein 1 OS=Sus scrofa OX=9823 GN=IQGAP1 PE=1 SV=1 |
| 11 | [Q29574_PIG](http://sale-depta-pc/mascot/cgi/master_results_2.pl?file=20190124%2FF003161.dat;pr.eh=11%2C11p;pr.page=11;pr.per_page=1;pr.show=proteins) | Histone H2B (Fragment) OS=Sus scrofa OX=9823 PE=2 SV=1 |
| 12 | [A0A287A096_PIG](http://sale-depta-pc/mascot/cgi/master_results_2.pl?file=20190124%2FF003161.dat;pr.eh=12%2C12p;pr.page=12;pr.per_page=1;pr.show=proteins) | Eukaryotic translation initiation factor 2 subunit 1 OS=Sus scrofa OX=9823 GN=EIF2S1 PE=1 SV=1 |
| 13 | [A0A287B3Y2_PIG](http://sale-depta-pc/mascot/cgi/master_results_2.pl?file=20190124%2FF003161.dat;pr.eh=13%2C13p;pr.page=13;pr.per_page=1;pr.show=proteins) | Non-POU domain containing octamer binding OS=Sus scrofa OX=9823 GN=NONO PE=1 SV=1 |
| 14 | [A0A287AB99_PIG](http://sale-depta-pc/mascot/cgi/master_results_2.pl?file=20190124%2FF003161.dat;pr.eh=14%2C14p;pr.page=14;pr.per_page=1;pr.show=proteins) | Keratin 12 OS=Sus scrofa OX=9823 GN=KRT12 PE=3 SV=1 |
| 15 | [F1S666_PIG](http://sale-depta-pc/mascot/cgi/master_results_2.pl?file=20190124%2FF003161.dat;pr.eh=15%2C15p;pr.page=15;pr.per_page=1;pr.show=proteins) | DExH-box helicase 9 OS=Sus scrofa OX=9823 GN=DHX9 PE=1 SV=3 |
| 16 | [A0A286ZYV1_PIG](http://sale-depta-pc/mascot/cgi/master_results_2.pl?file=20190124%2FF003161.dat;pr.eh=16%2C16p;pr.page=16;pr.per_page=1;pr.show=proteins) | Radixin OS=Sus scrofa OX=9823 GN=RDX PE=1 SV=1 |
| 17 | [A0A286ZTJ9_PIG](http://sale-depta-pc/mascot/cgi/master_results_2.pl?file=20190124%2FF003161.dat;pr.eh=17%2C17p;pr.page=17;pr.per_page=1;pr.show=proteins) | Sterol regulatory element-binding protein 1 OS=Sus scrofa OX=9823 GN=SREBF1 PE=4 SV=1 |
| 18 | [F1STE8_PIG](http://sale-depta-pc/mascot/cgi/master_results_2.pl?file=20190124%2FF003161.dat;pr.eh=18%2C18p;pr.page=18;pr.per_page=1;pr.show=proteins) | Eukaryotic translation initiation factor 5B OS=Sus scrofa OX=9823 GN=EIF5B PE=1 SV=3 |
| 19 | [A0A287BR84_PIG](http://sale-depta-pc/mascot/cgi/master_results_2.pl?file=20190124%2FF003161.dat;pr.eh=19%2C19p;pr.page=19;pr.per_page=1;pr.show=proteins) | Dolichyl-diphosphooligosaccharide--protein glycosyltransferase subunit 1 OS=Sus scrofa OX=9823 GN=R |
| 20 | [F1SKE7_PIG](http://sale-depta-pc/mascot/cgi/master_results_2.pl?file=20190124%2FF003161.dat;pr.eh=20%2C20p;pr.page=20;pr.per_page=1;pr.show=proteins) | Procollagen C-endopeptidase enhancer 2 OS=Sus scrofa OX=9823 GN=PCOLCE2 PE=4 SV=3 |
| 21 | [F1S895_PIG](http://sale-depta-pc/mascot/cgi/master_results_2.pl?file=20190124%2FF003161.dat;pr.eh=21%2C21p;pr.page=21;pr.per_page=1;pr.show=proteins) | Nucleolar protein 56 OS=Sus scrofa OX=9823 GN=NOP56 PE=1 SV=3 |
| 22 | [A0A287AL02_PIG](http://sale-depta-pc/mascot/cgi/master_results_2.pl?file=20190124%2FF003161.dat;pr.eh=22%2C22p;pr.page=22;pr.per_page=1;pr.show=proteins) | Non-specific serine/threonine protein kinase OS=Sus scrofa OX=9823 GN=AKT1 PE=4 SV=1 |
| 23 | [F1SDC2_PIG](http://sale-depta-pc/mascot/cgi/master_results_2.pl?file=20190124%2FF003161.dat;pr.eh=23%2C23p;pr.page=23;pr.per_page=1;pr.show=proteins) | Acid phosphatase 6, lysophosphatidic OS=Sus scrofa OX=9823 GN=ACP6 PE=1 SV=1 |
| 24 | [I3LIT1_PIG](http://sale-depta-pc/mascot/cgi/master_results_2.pl?file=20190124%2FF003161.dat;pr.eh=24%2C24p;pr.page=24;pr.per_page=1;pr.show=proteins) | Round spermatid basic protein 1 OS=Sus scrofa OX=9823 GN=RSBN1 PE=1 SV=2 |
| 25 | [ACTS_PIG](http://sale-depta-pc/mascot/cgi/master_results_2.pl?file=20190124%2FF003161.dat;pr.eh=25%2C25p;pr.page=25;pr.per_page=1;pr.show=proteins) | Actin, alpha skeletal muscle OS=Sus scrofa OX=9823 GN=ACTA1 PE=1 SV=1 |
| 26 | [A0A287BN04_PIG](http://sale-depta-pc/mascot/cgi/master_results_2.pl?file=20190124%2FF003161.dat;pr.eh=26%2C26p;pr.page=26;pr.per_page=1;pr.show=proteins) | Plectin OS=Sus scrofa OX=9823 GN=PLEC PE=1 SV=1 |
| 27 | [A0A287A536_PIG](http://sale-depta-pc/mascot/cgi/master_results_2.pl?file=20190124%2FF003161.dat;pr.eh=27%2C27p;pr.page=27;pr.per_page=1;pr.show=proteins) | Intersectin 1 OS=Sus scrofa OX=9823 GN=ITSN1 PE=1 SV=1 |
| 28 | [I3LU64_PIG](http://sale-depta-pc/mascot/cgi/master_results_2.pl?file=20190124%2FF003161.dat;pr.eh=28%2C28p;pr.page=28;pr.per_page=1;pr.show=proteins) | Protein phosphatase 1 regulatory subunit 3F OS=Sus scrofa OX=9823 GN=PPP1R3F PE=4 SV=2 |
| 29 | [A0A286ZYG8_PIG](http://sale-depta-pc/mascot/cgi/master_results_2.pl?file=20190124%2FF003161.dat;pr.eh=29%2C29p;pr.page=29;pr.per_page=1;pr.show=proteins) | Centrosomal protein 290 OS=Sus scrofa OX=9823 GN=CEP290 PE=4 SV=1 |
| 30 | [A0A287ATF1_PIG](http://sale-depta-pc/mascot/cgi/master_results_2.pl?file=20190124%2FF003161.dat;pr.eh=30%2C30p;pr.page=30;pr.per_page=1;pr.show=proteins) | Actin binding LIM protein family member 3 OS=Sus scrofa OX=9823 GN=ABLIM3 PE=1 SV=1 |
| 31 | [I3LH96_PIG](http://sale-depta-pc/mascot/cgi/master_results_2.pl?file=20190124%2FF003161.dat;pr.eh=31%2C31p;pr.page=31;pr.per_page=1;pr.show=proteins) | Family with sequence similarity 193 member A OS=Sus scrofa OX=9823 GN=FAM193A PE=4 SV=2 |
| 32 | [F1SUH8_PIG](http://sale-depta-pc/mascot/cgi/master_results_2.pl?file=20190124%2FF003161.dat;pr.eh=32%2C32p;pr.page=32;pr.per_page=1;pr.show=proteins) | V-type proton ATPase proteolipid subunit OS=Sus scrofa OX=9823 GN=ATP6V0C PE=1 SV=1 |
| 33 | [F1SAJ2_PIG](http://sale-depta-pc/mascot/cgi/master_results_2.pl?file=20190124%2FF003161.dat;pr.eh=33%2C33p;pr.page=33;pr.per_page=1;pr.show=proteins) | Uroplakin-2 OS=Sus scrofa OX=9823 GN=UPK2 PE=4 SV=3 |
| 34 | [A0A287AJH7_PIG](http://sale-depta-pc/mascot/cgi/master_results_2.pl?file=20190124%2FF003161.dat;pr.eh=34%2C34p;pr.page=34;pr.per_page=1;pr.show=proteins) | Autophagy related 101 OS=Sus scrofa OX=9823 GN=ATG101 PE=4 SV=1 |
| 35 | [I3L675_PIG](http://sale-depta-pc/mascot/cgi/master_results_2.pl?file=20190124%2FF003161.dat;pr.eh=35%2C35p;pr.page=35;pr.per_page=1;pr.show=proteins) | Myosin heavy chain 15 OS=Sus scrofa OX=9823 GN=MYH15 PE=3 SV=2 |
| 36 | [A0A0B8S015_PIG](http://sale-depta-pc/mascot/cgi/master_results_2.pl?file=20190124%2FF003161.dat;pr.eh=36%2C36p;pr.page=36;pr.per_page=1;pr.show=proteins) | Protein strawberry notch-like 1 OS=Sus scrofa domesticus OX=9825 GN=SBNO1 PE=4 SV=1 |
| 37 | [A0A287AL00_PIG](http://sale-depta-pc/mascot/cgi/master_results_2.pl?file=20190124%2FF003161.dat;pr.eh=37%2C37p;pr.page=37;pr.per_page=1;pr.show=proteins) | Regulator of G protein signaling 9 OS=Sus scrofa OX=9823 GN=RGS9 PE=1 SV=1 |
| 38 | [A0A287AN70_PIG](http://sale-depta-pc/mascot/cgi/master_results_2.pl?file=20190124%2FF003161.dat;pr.eh=38%2C38p;pr.page=38;pr.per_page=1;pr.show=proteins) | Uncharacterized protein OS=Sus scrofa OX=9823 GN=LOC100524970 PE=3 SV=1 |
| 39 | [A0A0B8S093_PIG](http://sale-depta-pc/mascot/cgi/master_results_2.pl?file=20190124%2FF003161.dat;pr.eh=39%2C39p;pr.page=39;pr.per_page=1;pr.show=proteins) | ATP-dependent RNA helicase DHX29 OS=Sus scrofa domesticus OX=9825 GN=DHX29 PE=3 SV=1 |
| 40 | [A0A287BKR3_PIG](http://sale-depta-pc/mascot/cgi/master_results_2.pl?file=20190124%2FF003161.dat;pr.eh=40%2C40p;pr.page=40;pr.per_page=1;pr.show=proteins) | Uncharacterized protein OS=Sus scrofa OX=9823 PE=4 SV=1 |
| 41 | [F1RGB5_PIG](http://sale-depta-pc/mascot/cgi/master_results_2.pl?file=20190124%2FF003161.dat;pr.eh=41%2C41p;pr.page=41;pr.per_page=1;pr.show=proteins) | Acetyl-CoA carboxylase 2 precursor OS=Sus scrofa OX=9823 GN=ACACB PE=4 SV=3 |
| 42 | [F1SBT5_PIG](http://sale-depta-pc/mascot/cgi/master_results_2.pl?file=20190124%2FF003161.dat;pr.eh=42%2C42p;pr.page=42;pr.per_page=1;pr.show=proteins) | General transcription factor IIF subunit 1 OS=Sus scrofa OX=9823 GN=GTF2F1 PE=4 SV=1 |
| 43 | [A0A287AI26_PIG](http://sale-depta-pc/mascot/cgi/master_results_2.pl?file=20190124%2FF003161.dat;pr.eh=43%2C43p;pr.page=43;pr.per_page=1;pr.show=proteins) | Uncharacterized protein OS=Sus scrofa OX=9823 PE=4 SV=1 |
| 44 | [A0A286ZIV9_PIG](http://sale-depta-pc/mascot/cgi/master_results_2.pl?file=20190124%2FF003161.dat;pr.eh=44%2C44p;pr.page=44;pr.per_page=1;pr.show=proteins) | Pre-mRNA-processing factor 40 homolog A OS=Sus scrofa OX=9823 GN=PRPF40A PE=1 SV=1 |
| 45 | [A0A287AAW3_PIG](http://sale-depta-pc/mascot/cgi/master_results_2.pl?file=20190124%2FF003161.dat;pr.eh=45%2C45p;pr.page=45;pr.per_page=1;pr.show=proteins) | Microtubule associated serine/threonine kinase family member 4 OS=Sus scrofa OX=9823 GN=MAST4 PE=4 |
| 46 | [A0A1D8MMA5_PIG](http://sale-depta-pc/mascot/cgi/master_results_2.pl?file=20190124%2FF003161.dat;pr.eh=46%2C46p;pr.page=46;pr.per_page=1;pr.show=proteins) | DEAD-box helicase 21 OS=Sus scrofa OX=9823 GN=DDX21 PE=2 SV=1 |
| 47 | [A0A287BE52_PIG](http://sale-depta-pc/mascot/cgi/master_results_2.pl?file=20190124%2FF003161.dat;pr.eh=47%2C47p;pr.page=47;pr.per_page=1;pr.show=proteins) | PC4 and SFRS1-interacting protein OS=Sus scrofa OX=9823 GN=PSIP1 PE=1 SV=1 |
| 48 | [K7GS03_PIG](http://sale-depta-pc/mascot/cgi/master_results_2.pl?file=20190124%2FF003161.dat;pr.eh=48%2C48p;pr.page=48;pr.per_page=1;pr.show=proteins) | LDL receptor related protein 1 OS=Sus scrofa OX=9823 GN=LRP1 PE=1 SV=2 |
| 49 | [A0A287BNT3_PIG](http://sale-depta-pc/mascot/cgi/master_results_2.pl?file=20190124%2FF003161.dat;pr.eh=49%2C49p;pr.page=49;pr.per_page=1;pr.show=proteins) | Uncharacterized protein OS=Sus scrofa OX=9823 PE=4 SV=1 |
| 50 | [A0A286ZRX6_PIG](http://sale-depta-pc/mascot/cgi/master_results_2.pl?file=20190124%2FF003161.dat;pr.eh=50%2C50p;pr.page=50;pr.per_page=1;pr.show=proteins) | Platelet endothelial aggregation receptor 1 OS=Sus scrofa OX=9823 GN=PEAR1 PE=4 SV=1 |
| 51 | [F1SRI2_PIG](http://sale-depta-pc/mascot/cgi/master_results_2.pl?file=20190124%2FF003161.dat;pr.eh=51%2C51p;pr.page=51;pr.per_page=1;pr.show=proteins) | Transcription elongation factor A N-terminal and central domain containing OS=Sus scrofa OX=9823 GN |
| 52 | [F1RZQ7_PIG](http://sale-depta-pc/mascot/cgi/master_results_2.pl?file=20190124%2FF003161.dat;pr.eh=52%2C52p;pr.page=52;pr.per_page=1;pr.show=proteins) | Prefoldin subunit 3 OS=Sus scrofa OX=9823 GN=VBP1 PE=1 SV=3 |
| 53 | A0A4X1VIP2_PIG | Uncharacterized protein OS=Sus scrofa OX=9823 GN=SYNCRIP PE=4 SV=1 |
| 54 | [A0A287AYP4_PIG](http://sale-depta-pc/mascot/cgi/master_results_2.pl?file=20190124%2FF003161.dat;pr.eh=53%2C53p;pr.page=53;pr.per_page=1;pr.show=proteins) | Uncharacterized protein OS=Sus scrofa OX=9823 GN=FAM208B PE=4 SV=1 |
| 55 | [A0A286ZVK7_PIG](http://sale-depta-pc/mascot/cgi/master_results_2.pl?file=20190124%2FF003161.dat;pr.eh=54%2C54p;pr.page=54;pr.per_page=1;pr.show=proteins) | Phosphatidylinositol-4,5-bisphosphate 3-kinase catalytic subunit alpha OS=Sus scrofa OX=9823 GN=PIK |
| 56 | [F1RJP0_PIG](http://sale-depta-pc/mascot/cgi/master_results_2.pl?file=20190124%2FF003161.dat;pr.eh=55%2C55p;pr.page=55;pr.per_page=1;pr.show=proteins) | Chromosome 8 open reading frame 74 OS=Sus scrofa OX=9823 GN=C8orf74 PE=4 SV=2 |
| 57 | [A0A287A1N6_PIG](http://sale-depta-pc/mascot/cgi/master_results_2.pl?file=20190124%2FF003161.dat;pr.eh=56%2C56p;pr.page=56;pr.per_page=1;pr.show=proteins) | DNA-directed RNA polymerase III subunit RPC6 OS=Sus scrofa OX=9823 GN=POLR3F PE=3 SV=1 |
| 58 | [F1RLQ7_PIG](http://sale-depta-pc/mascot/cgi/master_results_2.pl?file=20190124%2FF003161.dat;pr.eh=57%2C57p;pr.page=57;pr.per_page=1;pr.show=proteins) | Uncharacterized protein OS=Sus scrofa OX=9823 GN=CCDC168 PE=4 SV=3 |
| 59 | [A0A287BFK8_PIG](http://sale-depta-pc/mascot/cgi/master_results_2.pl?file=20190124%2FF003161.dat;pr.eh=58%2C58p;pr.page=58;pr.per_page=1;pr.show=proteins) | Uncharacterized protein OS=Sus scrofa OX=9823 PE=4 SV=1 |
| 60 | [F1RV90_PIG](http://sale-depta-pc/mascot/cgi/master_results_2.pl?file=20190124%2FF003161.dat;pr.eh=59%2C59p;pr.page=59;pr.per_page=1;pr.show=proteins) | Male germ cell associated kinase OS=Sus scrofa OX=9823 GN=MAK PE=1 SV=3 |
| 61 | [A0A286ZIM7_PIG](http://sale-depta-pc/mascot/cgi/master_results_2.pl?file=20190124%2FF003161.dat;pr.eh=60%2C60p;pr.page=60;pr.per_page=1;pr.show=proteins) | Protein NDRG2 OS=Sus scrofa OX=9823 GN=NDRG2 PE=1 SV=1 |
| 62 | [A0A287AYU1_PIG](http://sale-depta-pc/mascot/cgi/master_results_2.pl?file=20190124%2FF003161.dat;pr.eh=61%2C61p;pr.page=61;pr.per_page=1;pr.show=proteins) | Metastasis associated 1 family member 2 OS=Sus scrofa OX=9823 GN=MTA2 PE=1 SV=1 |
| 63 | [A0A287A2D8_PIG](http://sale-depta-pc/mascot/cgi/master_results_2.pl?file=20190124%2FF003161.dat;pr.eh=62%2C62p;pr.page=62;pr.per_page=1;pr.show=proteins) | Myosin IXA OS=Sus scrofa OX=9823 GN=MYO9A PE=3 SV=1 |
| 64 | [F1S8S9_PIG](http://sale-depta-pc/mascot/cgi/master_results_2.pl?file=20190124%2FF003161.dat;pr.eh=63%2C63p;pr.page=63;pr.per_page=1;pr.show=proteins) | Golgi brefeldin A resistant guanine nucleotide exchange factor 1 OS=Sus scrofa OX=9823 GN=GBF1 PE=1 |
